# Sumatriptan Induced Takotsubo Cardiomyopathy; the Headache of the Heart: A Case Report

**DOI:** 10.3389/fcvm.2019.00134

**Published:** 2019-09-18

**Authors:** Jay Mohan, Akarsh Parekh, Michael DeYoung

**Affiliations:** ^1^Department of Cardiology, McLaren Macomb Regional Medical Center, Mount Clemens, MI, United States; ^2^Department of Internal Medicine, McLaren Macomb Regional Medical Center, Mount Clemens, MI, United States

**Keywords:** Takotsubo Cardiomyopathy, cardiomyopathy, stress-induced cardiomyopathy, broken heart syndrome, sumatriptan, triptans, headaches, migraines

## Abstract

Takotsubo Cardiomyopathy (TCM) is an increasing recognized form of acute reversible left ventricular systolic dysfunction not related to obstructive coronary disease. The exact physiology of this disorder is not yet known, however multiple agents have been hypothesized to have a link to this condition. Most commonly, TCM has been hypothesized as being triggered by a catecholamine surge after an inciting event. New evidence now suggests certain medications as a link to the disease. We describe a unique case of TCM in a woman after taking Treximet (naproxen and sumatriptan) as abortive therapy for a migraine.

## Background

Takotsubo Cardiomyopathy is an increasing recognized form of acute reversible left ventricular systolic dysfunction. It is mainly recognized as a stress induced myopathy that is usually caused by physical or psychological distress. Merchant et al. describes it as “transient left ventricular (LV) apical ballooning syndrome,” “takotsubo-like left ventricular dysfunction,” “ampulla cardiomyopathy,” “stress-induced cardiomyopathy,” and “broken heart syndrome” (1). In Japanese, “tako tsubo” translates to “octopus pot,” which is a fishing jar with a narrow neck and wide base used to trap octopus, and describes the visual appearance of the heart on left ventriculography (1). This cardiomyopathy usually presents with classic signs and symptoms of acute coronary syndrome with dyspnea, chest pain, elevated cardiac biomarkers, and electrocardiographic changes suggestive of ischemia. It is most often characterized with the use of coronary angiography that demonstrates little to no significant obstructive atherosclerotic coronary vascular disease and classic apical ballooning seen on left ventriculography.

Most commonly, TCM has been hypothesized as being triggered by a catecholamine surge after an inciting event. Numerous other triggers have been postulated in the literature. We describe a unique case of TCM in a woman after taking Treximet (sumatriptan and naproxen) as abortive therapy for a migraine.

## Case Presentation

**Figure 1:**
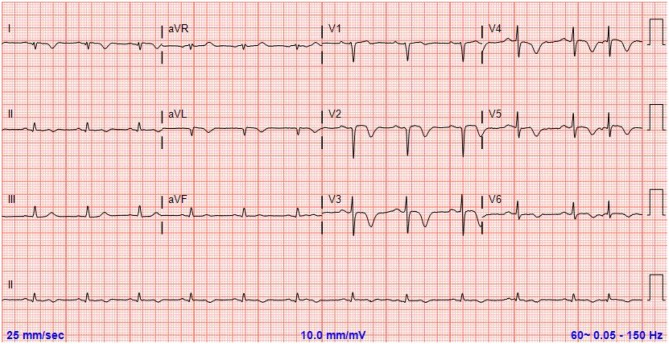
Electrocardiograme demonstrating symmetrical T wave inversions in the anterolateral leads concerning for ischemia.

**Figure 2:**
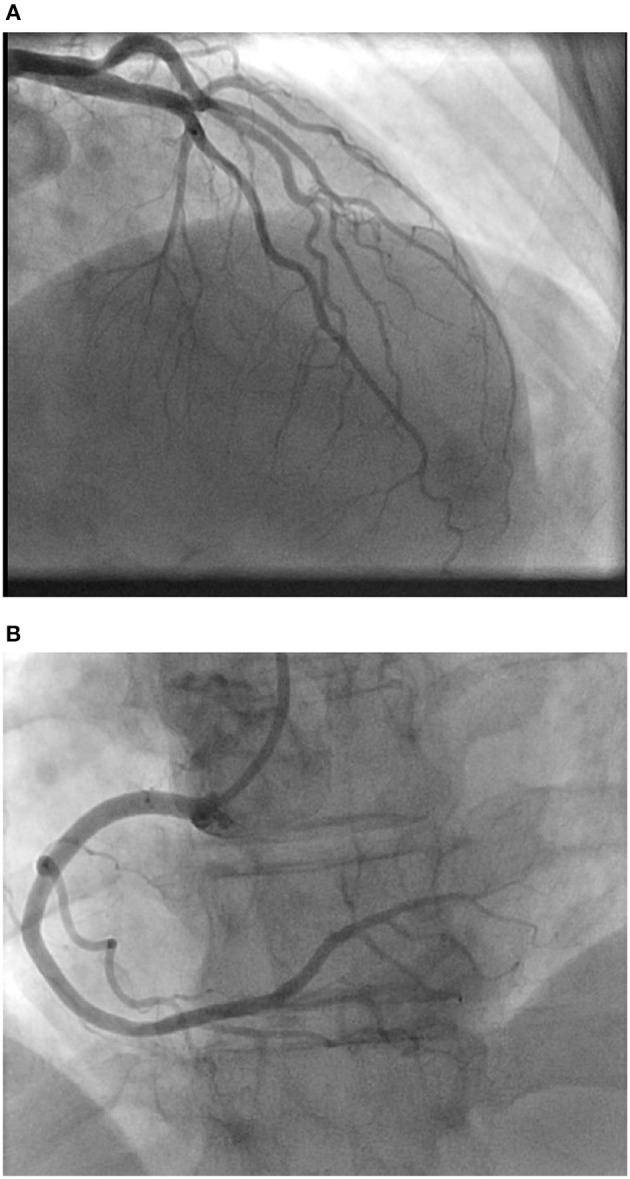
**(A)** Coronary angiogram showing left coronary artery circulation with a non-occlusive lesion in proximal left circumflex artery. **(B)** Coronary angiogram showing right coronary artery circulation.

**Figure 3:**
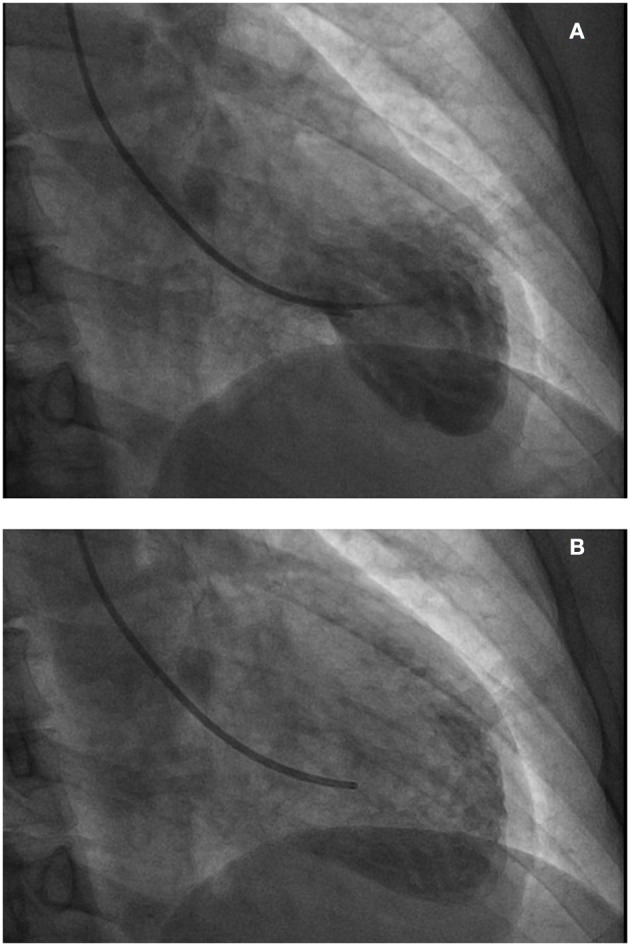
**(A,B)** Left ventriculography demonstrating apical ballooning and hypokinesis of the apex with normal functioning basal inferior and anterior segments.

A 59 year-old Caucasian female with past medical history of migraines, presented to our emergency department as a transfer from another facility for further management of a non-ST elevation myocardial infarction. In the emergency room she presented with notable anterolateral T wave inversions on ECG as well as elevated troponin I of 2.5 ng/mL (reference range: 0.000–0.039 ng/mL). vThe patient was having continued chest pain at time of arrival and stated that she experienced her first acute episode of chest pain 1 day earlier, occurring at her home, while at rest. She stated that earlier in the day she was experiencing a migraine and took her home medication of Treximet 85/500 mg with good relief of her symptoms. The patient stated that she occasionally gets migraines and on occasion uses Treximet to relieve her symptoms, however has not had to take the medication in quite some time. The patient claimed that a few hours after her migraine had resolved she began to experience non-radiating, left sided, substernal chest pressure with associated paresthesia in the lower extremities and some non-descriptive nausea. This prompted her to seek medical attention at a local urgent care facility. She was seen and assessed by a physician and had an ECG performed which demonstrated no significant issues so she was discharged home with an antacid and told to follow up with her primary physician as an outpatient. The patient stated that the chest pain never really dissipated however she went to sleep that night without issues. The next morning, she woke up with continued chest pressure, yet, decreased in quality from the previous day. She went to work that day and while talking to her coworkers she immediately felt worsening of her chest pressure and was taken to a local hospital, without catheterization capabilities, where she was noted to have new ECG changes and elevated troponins. Upon transfer to our facility she was given 325 mg of aspirin by mouth and started on IV heparin and nitroglycerine. ECGs at our hospital showed progression of her anterolateral T wave inversions (see Figure 1) therefore it was deemed necessary to take the patient for urgent coronary angiography with possible percutaneous intervention to evaluate for obstructive coronary artery disease. The patient was taken to the cardiac catheterization laboratory and successfully underwent a left heart catheterization with selective left and right coronary angiogram (see Figures 2A,B) which did not reveal any significant obstructive atherosclerotic coronary artery stenosis; except a non-occlusive lesion in left circumflex artery which was not significant enough for any intervention and less likely to cause our patients acute symptoms and echocardiographic findings. Left ventriculography performed at the time demonstrated notable apical ballooning and hypokinesis of the apex with normal functioning basal, anterior, lateral, and inferior walls (see Figures 3A,B) as well as an ejection fraction estimated at 30% consistent with TCM.

**Figure 4:**
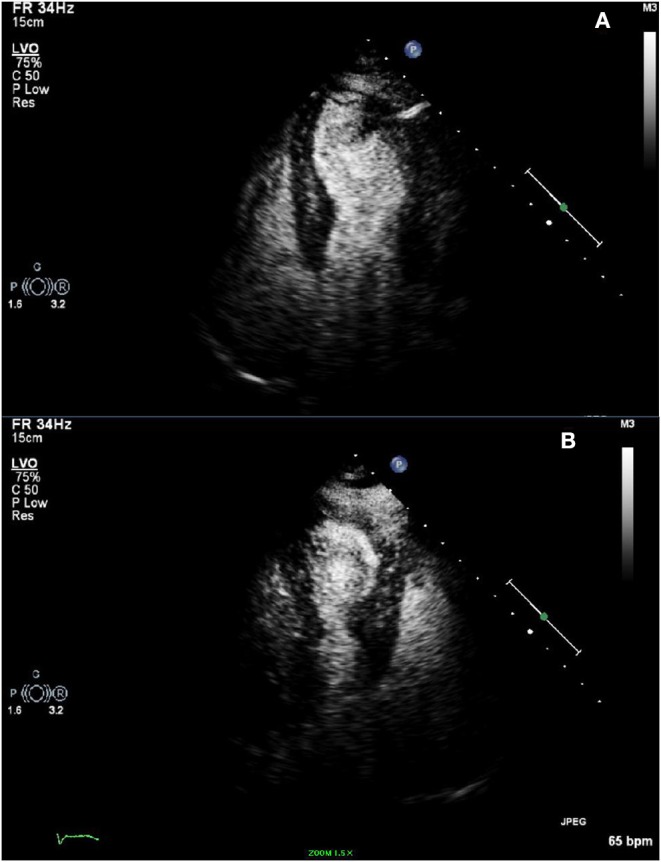
**(A,B)** Transthoracic echocardiogram with definity contrast demonstrating hypercontractility of the basal wall and akinesis of the mid to distal anteroseptal wall, anterior wall, mid to distal inferior and inferolateral walls, and apex.

The patient had no post procedural complications and chest pain had since resolved. Her echocardiogram post-cardiac catheterization reported a left ventricular ejection fraction on 30% with hypercontractility of the basal wall and akinesis of the mid to distal anteroseptal wall, anterior wall, mid to distal inferior and inferolateral walls, and apex consistent with TCM (see Figures 4A,B). Patient was placed on metoprolol succinate 25 mg once a day, advised to avoid Treximet or other migraine medications if possible, and was informed to follow up in the office as an outpatient for a follow up echocardiogram. Patient remained symptom free and had a follow up echocardiogram done 3 months post discharge, which demonstrated resolution of her wall motion abnormalities and improvement of her ejection fraction to 55%.

## Discussion

As medicine, in particular, cardiology continues to evolve, the detection of syndromes like TCM will continue to emerge. Once thought of as an exceedingly rare etiology, the diagnosis of TCM has steadily increased as our knowledge of the disease continues to progress. Akashi et al. suggests that between 1989 and December 2007 there has been an exponentially increasing frequency of publications regarding TCM (2). Until 2000, a few case reports were published, but the presentation of Takotsubo Cardiomyopathy has increased gradually since 2001. The number of publications has increased rapidly reflected the idea that TCM has probably gained broad attention in the field of cardiology among US and European physicians (2).

The true prevalence of this cardiomyopathy is unclear and there are no clear risk factors associated with TCM (3). On the basis of recent analyses reported from several countries, this condition probably accounts for 1–2% of all cases of suspected acute myocardial infarction (4). There currently is no consensus on the diagnostic criteria for TCM. Per Akashi et al. researchers at the Mayo Clinic proposed diagnostic criteria in 2004, which have been modified recently: (i) transient hypokinesis, akinesis, or dyskinesis in the left ventricular mid segments with or without apical involvement; regional wall motion abnormalities that extend beyond a single epicardial vascular distribution; and frequently, but not always, a stressful trigger; (ii) the absence of obstructive coronary disease or angiographic evidence of acute plaque rupture; (iii) new ECG abnormalities (ST-segment elevation and/or T-wave inversion) or modest elevation in cardiac troponin; and (iv) the absence of pheochromocytoma and myocarditis (2).

Although the clinical presentation of TCM appears very similar to acute coronary syndrome the pathophysiology of the two seem quite different. Merchant et al. describes three proposed mechanisms of the disease: (i) multivessel coronary artery spasm, (ii) impaired cardiac microvascular function, and (iii) endogenous catecholamine induced myocardial stunning and microinfarction (1). With the elevation of cardiac biomarkers, the idea of myocardial ischemia must be entertained. Whether this is caused by microvascular disease or actual occlusion of a major epicardial vessel is unknown. Further analyzing the histology from myocardial biopsy samples, the inflammatory changes in TCM differ from coagulation necrosis as seen in myocardial infraction from coronary artery occlusion (2, 5). This gives rise to the idea that inflammation from another acute process is the causative agent rather than coronary occlusion. The most postulated cause of TCM revolves around the idea of a sudden catecholamine surge that possibly leads to coronary vasospasm and neuromodulators dysfunction leading to increased free radical release (6). Nef et al. describes this mechanism in stating that catecholamine produces vasoconstriction and direct myocyte damage from increased calcium release, which activates cAMP, causing myocyte damage and free radical release (6). This may be the mechanism that is responsible for the possible myocardial stunning that we see with TCM.

Our patient had taken Treximet (sumatriptan and naproxen) for her headaches. Acute migraines in themselves can cause a sudden catecholamine surge, however neither migraines nor naproxen use are well-reported as a causative agent in TCM. Rather there are limited case reports of TCM post migraine abortion with triptan medications, particularly sumatriptan. The mechanism of action of sumatriptan revolves around its selective serotonin agonistic properties and its ability to cause activation of vascular serotonin 5-HT1 receptors, which in turn, causes vasoconstriction (7). Although not proven, theoretically sumatriptan can induce coronary vasospasm, usually in a situation where there is already heightened sympathetic drive and increase neuromodulation in the setting of an acute migraine. Currently there are only a few case reports of triptan induced cardiomyopathy (8, 9). For that reason, further research is needed to confirm a true cause and effect relationship between these two entities (1, 8).

## Data Availability

All datasets generated for this study are included in the manuscript/supplementary files.

## Consent

A signed informed written informed consent was obtained from the participant for the publication of this case report.

## Ethics Statement

Ethical review and approval was not required for the study on human participants in accordance with the local legislation and institutional requirements. The patients/participants provided their written informed consent to participate in this study.

## Author Contributions

JM contributed in literature review, case presentation, composing, and editing the manuscript. AP and MD assisted in manuscript editing.

### Conflict of Interest Statement

The authors declare that the research was conducted in the absence of any commercial or financial relationships that could be construed as a potential conflict of interest.
